# The effect of rule retrieval on activity in the default mode network

**DOI:** 10.1016/j.neuroimage.2019.116088

**Published:** 2019-11-15

**Authors:** Verity Smith, Daniel J. Mitchell, John Duncan

**Affiliations:** a Medical Research Council Cognition and Brain Sciences Unit, United Kingdom; b University of Cambridge, United Kingdom; c University of Oxford, United Kingdom

**Keywords:** fMRI, Cognitive control, DMN

## Abstract

The default mode network (DMN) is often associated with internally-directed cognition, distinct from the constraints of the external environment. However, a recent finding is that the DMN shows strong activation after large task switches during a demanding externally-directed task (Crittenden et al., 2015; Smith et al., 2018). Following other proposals, we have suggested that the DMN encodes cognitive or environmental context, and that context representations are momentarily strengthened during large cognitive switches, perhaps so that new activity can be checked against current environmental constraints. An alternative account, consistent with the role of the DMN in episodic memory, might be that switches to a substantially new task increase demands on rule retrieval. To test this alternative, we directly manipulated rule retrieval demands. Contrary to the retrieval account, increased retrieval demand led to reduced DMN activity, accompanied by increased activation in prefrontal and lateral parietal cognitive control areas. Unlike episodic retrieval, with its rich contextual representations, rule retrieval does not drive DMN activity. Accordingly, it cannot explain increased DMN activity during large cognitive switches.

## Highlights.

• Rule retrieval demand is associated with deactivation of default mode (DM) regions.
• Thus DM activity at large task switches is unlikely to be due to rule retrieval.
• DM activity in response to task switches may depend on hierarchical task structure.

## 1 Introduction

The default mode network (DMN) is a set of brain regions including parts of the posterior cingulate, medial prefrontal, retrosplenial, medial temporal, lateral temporal and inferior parietal cortices. The DMN is one of the best established brain networks, with its emergence consistently replicated through functional connectivity methods (Greicius et al., 2003, 2009; Spreng et al., 2013; Liang et al., 2015). Early research characterised the DMN as “task negative”, with deactivations during many externally-focused tasks in comparison to rest or easier versions of the same task (Shulman et al., 1997; McKiernan et al., 2003). DMN activity has also frequently been associated with off-task thinking or mind wandering (McKiernan et al., 2006; Mason et al., 2007; Christoff et al., 2009). In line with these task-related deactivations, the DMN is often anti-correlated with regions associated with on-task executive function (Fox et al., 2005a; Kelly et al., 2008; Uddin et al., 2009; Newton et al., 2011).

Since these early findings, research has shown overlapping DMN activation in a diverse set of tasks (Spreng et al., 2009). DMN regions have been linked to a number of aspects of episodic memory retrieval including of memory for contextual details of remembered items (Hayama et al., 2012; Vilberg and Rugg, 2012). DMN regions have also been implicated in imagining personal events relating to past, present or future (Addis et al., 2007; Andrews-Hanna et al., 2010). Other research has highlighted the importance of the DMN in social cognition (Frith and Frith, 2003), scene construction (Baldassano et al., 2016; Robin et al., 2018) and spatial navigation (Schinazi and Epstein, 2010; Balaguer et al., 2016). Attempts to understand a common DMN function during these cognitive processes broadly suggest that the DMN is important for some aspect of internal mentation, where cognition is not constrained by what is perceptually present (Buckner et al., 2008; Andrews-Hanna, 2012). Possibilities include imagination of internally-generated scenes, as well as self-relevant cognition (Hassabis and Maguire, 2007; Buckner and Carroll, 2007).

Evidence is now emerging, however, to show DMN activation during some kinds of complex, externally-directed activity. In two recent studies (Crittenden et al., 2015; Smith et al., 2018), participants saw stimuli from three different domains (e.g. faces, buildings and letter strings), and were cued to perform one of six different tasks (two tasks per domain). The task to carry out was cued by the colour of a surrounding frame, presented either simultaneously with or in advance of the primary stimulus. Following Andrews-Hanna (2012), the DMN was divided into 3 subnetworks – “core” (anteromedial prefrontal, posterior cingulate), “MTL” (posterior parietal, hippocampual/parahippocampal, retrosplenial, ventromedial prefrontal), and “dmPFC” (dorsomedial prefrontal, temporoparietal junction, lateral temporal, temporal pole). For core and MTL subnetworks, switching to tasks of a different domain was found to show increased activation compared to switching to a task of the same stimulus domain or repeating the same task. Perhaps most interestingly, the design of Smith et al. (2018) also included occasional “rest” trials. As expected, activity in core and MTL subnetworks increased with a switch from task to rest, but also with a switch from rest back to task (see also Fox et al., 2005b). To explain these findings, Smith et al. (2018) followed the evidence suggesting that the DMN – at least the core and MTL components – represents cognitive contexts or situation models (Zacks et al., 2007; Bar, 2007, 2009; Ranganath and Ritchey, 2012). Such contexts might include spatial, temporal, social and perhaps other descriptions of a cognitive environment. Though many previous accounts have emphasized internally-generated contexts, such as the setting for an autobiographical memory, Smith et al. suggested that contexts could be either internally created or imposed by the environment. To explain their findings, Smith et al. (2018) suggested that, through a series of similar task trials, representation of the broader context or environment progressively fades, as attention is increasingly focused on the task at hand. With switch to a substantially new line of activity, however, representation of the broader context is reinstated, perhaps because new activity should be checked against current environmental constraints.

Here we test an alternative account of the Crittenden et al. (2015) and Smith et al. (2018) findings. Though DMN activity is well known in the context of episodic recollection, some authors have proposed a broader role in information retrieval. Spreng et al. (2014) found increases in DMN activity during a 2-back working memory task using famous faces compared to anonymous faces, suggesting that the DMN contributes to task performance when this requires retrieval from long term knowledge. In a series of studies, Smallwood et al. found increased DMN activation when participants were required to make decisions about stimuli presented in the previous trial compared to perceptual decisions about current stimuli (Smallwood et al., 2013; Konishi et al., 2015; Murphy et al., 2018). They suggest that DMN activations during externally-directed tasks are due to retrieval of information which is not available in the current environment. Along similar lines, it could be argued that DMN activation during transitions to more dissimilar tasks in Crittenden et al. (2015) and Smith et al. (2018) is simply an effect of memory retrieval demand. For example, in repeat trials, participants simply have to retrieve the same colour-rule information as retrieved on the previous trial. Retrieval might also be easy on within-domain switch trials if participants have formed a strong association between the two colours associated with the same stimulus domain. In this case, when the colour switches but the stimulus domain remains the same, participants can simply switch to the other rule of this domain. Retrieval demands are likely higher on between-domain switch trials and restarts following rest, where such short-cuts are not possible.

In the previous studies (Crittenden et al., 2015; Smith et al., 2018) effects of rule retrieval difficulty and degree of switch were inseparable. In the current study we aimed to examine rule retrieval directly. To this end, we manipulated retrieval demand by varying the number of alternative rules in the task set. Additionally, we asked whether switches in stimulus domain continue to increase DMN activity in a setting substantially simpler than the Crittenden et al. (2015) and Smith et al. (2018) tasks. We compared activity in the three sub-networks of the DMN, with a set of typically task-related “multiple-demand” (MD) regions. These regions typically show increased activity with increased task difficulty (Duncan and Owen, 2000; Duncan, 2013; Fedorenko et al., 2013) and include parts of the inferior frontal sulcus, dorsal prefrontal cortex, inferior frontal junction, anterior insula, presupplementary motor area and intraparietal sulcus. Against the suggestion that activity might reflect rule retrieval, our results show increased MD activity but decreased DMN activity with increased retrieval demand.

## 2 Methods

### 2.1 Participants

46 participants (27 female), between 18 and 35 years old, were recruited through the Medical Research Council Cognition and Brian Sciences Unit volunteer panel. All participants selected were right handed, native English speakers, with normal or corrected to normal vision, and between 18 and 40 years old. The experiment was con-ducted in accordance with ethics approval granted from the Cambridge Psychology Research Ethics Committee. 4 participants (2 female) were excluded from further analysis due to poor task performance (n = 1), excessive motion (n = 2) or mid-task cancellation (n = 1).

### 2.2 Task

**Fig. 1:**
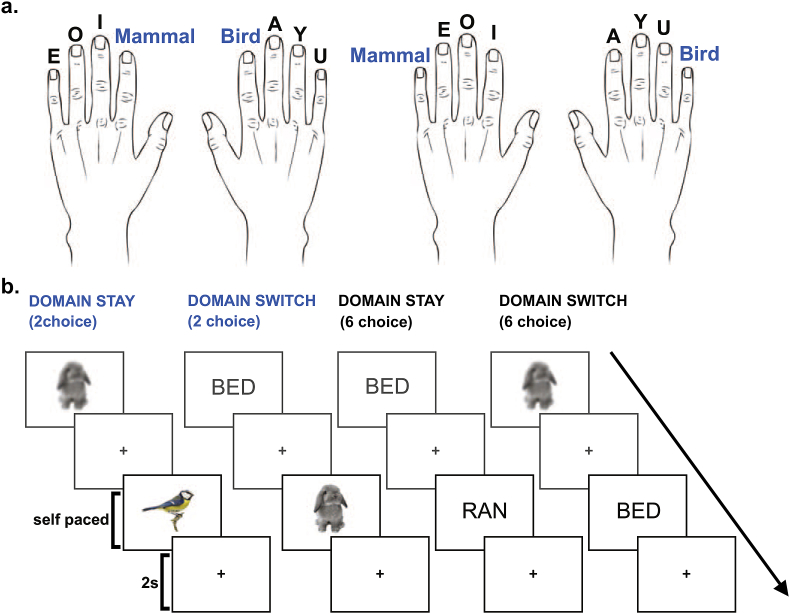
Task design, illustrated for the group with a 6-choice word task. a. The two possible category-response mappings. b. Example trials for each combination of choice number (2 or 6) by domain switch condition (switch or stay). Each trial was followed by a 2 s inter-trial interval.

Task events are illustrated in Fig. 1. Participants were presented with either written words, or pictures of animals, and asked to press one of 8 buttons depending on the category of item that was presented. Half (21) of the participants were asked to classify 6 types of animal (bird, fish, insect, mammal, mollusc, reptile) and identify which of 2 vowels was used in the word stimuli (A, E). The other (21) participants had to classify 2 types of animal (bird, mammal) and identify which of 6 vowels was used in the word stimuli (A,E,I,O,U,Y). Within each group, the response mappings were counterbalanced such that 2-choice responses were made with index fingers for half of the participants, and with little fingers for the other half. Fig. 1a shows the possible response mappings for the group with a 6-choice word task.

Each stimulus remained until a button press was made. Participants were asked to respond as quickly as possible without making mistakes. An inter-trial interval (ITI) of 2 s followed each response, with a fixation cross presented in the centre of the screen.

The two stimulus domains were presented in pseudorandom order. This generated four conditions that crossed the number of response options (2 or 6) with whether the stimulus domain repeated or switched: 2-choice domain-stay (a 2-choice stimulus following another), 2-choice domain-switch (switch from a 6-choice stimulus to a 2-choice stimulus), 6-choice domain-stay (a 6-choice stimulus following another) and 6-choice domain-switch (switch from a 2-choice stimulus to a 6-choice stimulus). The experiment consisted of a single block of 145 trials. Each block contained 36 trials of each condition, plus the first trial of the block (switch type undefined) which was discarded from further analysis. In Table 1, the total number of analysed trials is further broken down by whether, on successive trials, responses were the same (response stay), different but from the same hand (hand stay), or from different hands (hand switch).

**Table 1:** Number of trials per response choice number, domain switch type and response switch type.

		Response Stay	Hand Stay	Hand Switch
2 Choice	Domain Stay	18	0	18
	Domain Switch	0	18	18
6 Choice	Domain Stay	6	12	18
	Domain Switch	0	18	18

The experiment was controlled using Psychophysics Toolbox for MATLAB (Brainard and Vision, 1997). Stimuli were presented on a screen located at the back of the scanner, made visible to participants via a mirror mounted on a 32 channel head coil. All stimuli were presented on a white background in the centre of the screen, sized to fit snugly inside a rectangle measuring approximately 6.0 (width) x 4.5 (height) degrees of visual angle. The word stimuli were presented in black upper case and varied from 3 to 6 letters long. All word stimuli and picture stimuli were chosen to be familiar and recognisable to the participants as developed through behavioural pilots. For each participant there was a pool of 48 word stimuli and 48 picture stimuli. There were 24 stimuli for each of the 2 choice categories and 8 stimuli for each of the 6 choice categories. For example, for a participant with the 6 choice word task there would be 24 mammal pictures, 24 bird pictures, 8 A-words, 8 E-words, 8 I-words, 8 O-words, 8 U-words and 8 Y-words. Within each stimulus category, the exact stimulus presented on a given trial was selected at random but always different from the previously presented stimulus.

### 2.3 Training

Participants were carefully pre-trained to ensure good learning of button presses. To encourage separation of the two task domains, participants were first presented with the 2-choice response rules and then asked to repeat them from memory. Next, participants were presented with the 6-choice response rules and asked to repeat them from memory. After learning the 2-choice and 6-choice rules separately, participants were shown one exemplar from each response category in a random order and asked which finger they would use to respond. This process was repeated twice. At the second run-through, all participants got all responses correct. Finally, participants performed a short practice block of 36 trials (9 from each combination of 2/6 choice x domain stay/switch) outside the scanner.

### 2.4 Data acquisition

Images were acquired using a 3 ​T ​S Prisma magnetic resonance imaging (MRI) scanner, fitted with a 32-channel head coil. Functional MRI (fMRI) acquisitions used T2*-weighted multiband Echo-Planar Imaging (multiband acquisition factor 3 for 2.5 ​mm slices with no interslice gap, TR 1.1 ​s, TE 30 ​ms, flip angle 62°, voxel size 2 ​× ​2 ​mm in plane). T1-weighted multiecho magnetization-prepared rapid gradient-echo (MPRAGE) images were also obtained (TR 2.53 ​s, TE 1.64, 3.50, 5.36 and 7.22 ​ms, flip angle 9°, voxel size 1 mm3).

### 2.5 Preprocessing

Images were preprocessed using automaticanalysis (version 4) (Cusack et al., 2015) and SPM 12 (Wellcome Department of Cognitive Neurology, London, UK) for Matlab (Mathworks). The sequence of preprocessing stages involved spatial realignment of the raw EPIs, slice-time correction to the middle slice, coregistration of the functional EPI images to the structural T1-weighted image, and normalization to the Montreal Neurological Institute (MNI) template brain. To match Smith et al. (2018), functional images were then spatially smoothed using a Gaussian kernel of 10 mm full-width at half-maximum.

### 2.6 Regions of interest

**Fig. 2:**
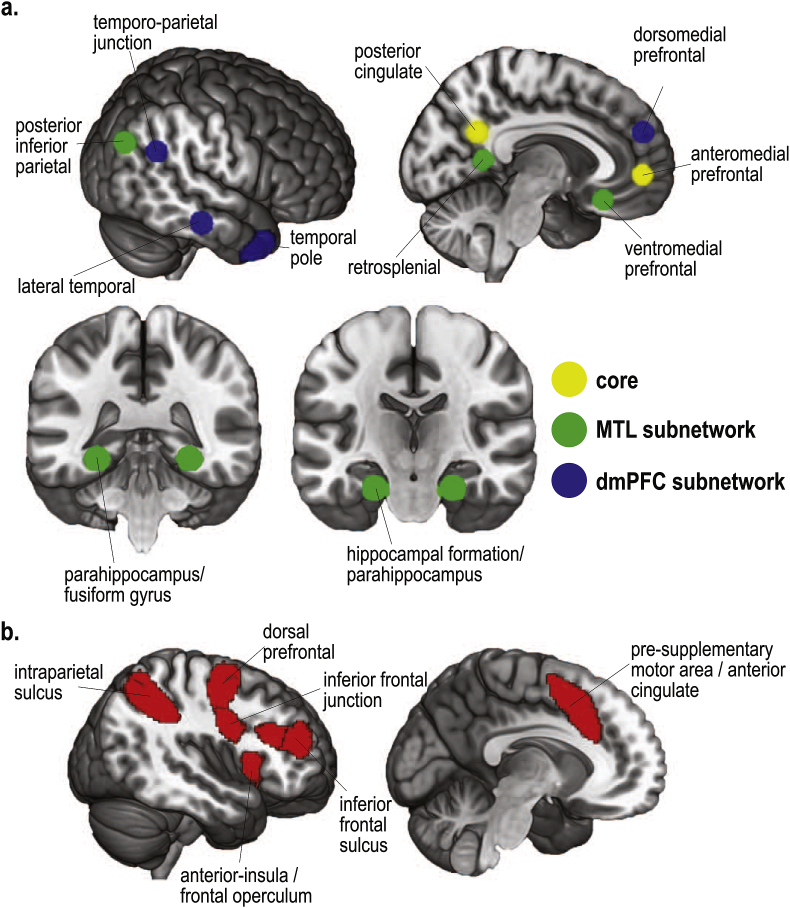
Regions of interest. a. DMN ROIs from peak coordinates presented in Andrews-Hanna et al. (2010). b. MD ROIs from Fedorenko et al. (2013). Figure taken from Smith et al. (2018).

To stay as close as possible to Smith et al. (2018) we used the same ROIs, presented in Fig. 2. The DMN ROIs (Fig. 2a) are comprised of 8 mm radius spheres around peak coordinates from Andrews-Hanna et al. (2010). The DMN ROIs are clustered into core, MTL and dmPFC subnetworks based on the functional connectivity and univariate activity results from Andrews-Hanna et al. (2010). Consistent with Smith et al. (2018), due to the position of the bounding box, some voxels surrounding the Andrews-Hanna et al. (2010) temporal pole peak were not measured; to compensate for this, the temporal pole ROIs were each expanded in radius by 2 voxels. Frontoparietal MD ROIs (Fig. 2b) were taken from Fedorenko et al. (2013), including the posterior–anterior extent of the inferior frontal sulcus, dorsal prefrontal cortex, inferior frontal junction, anterior insula/frontal operculum, presupplementary motor area/dorsal anterior cingulate, and intraparietal sulcus. Volumes were down-loaded from http://imaging.mrc-cbu.cam.ac.uk/imaging/Mdsystem using the separate-ROI version and selecting only the frontoparietal ROIs.

### 2.7 Analysis

fMRI data for each participant were examined using the General Linear Model. Regressors were separately created for each combination of choice number (6, 2) by domain switch condition (domain stay, domain switch) by response switch type (response stay, hand stay, hand switch). Each regressor was modelled as a rectangular function from stimulus onset to response, convolved with the canonical hemodynamic response function. Given that participant accuracy was very high (mean 98.3%), the original GLM did not exclude error trials. To check for any effect of error trials, in a follow-up GLM (not presented) each error trial was modelled separately and excluded from further analysis. This model produced almost identical results to those presented here.

Beta weight images were subtracted for the contrasts 6-choice>2-choice, 2-choice>6-choice, domain switch > domain stay, and domain stay > domain switch. As shown in Table 1, there were no domain-switch, response-stay trials, or 2-choice, domain-stay, hand-stay trials, and therefore no regressors for these conditions. We took a number of steps in order to ensure that each contrast was balanced accordingly. For the contrast of 6-choice>2-choice (and 2-choice>6-choice) we chose to exclude domain-stay, hand-stay, 6-choice trials, averaging regressors for the remaining 4 trial types in 2- and 6- choice conditions (Table 1). To examine domain switch effects, we used only hand switch trials as these were matched for 2- and 6- choice (Table 1). To check on domain switch effects specific to a response choice condition, we also ran switch contrasts separately for 2- and 6-choice regressors.

For the ROI analysis, mean contrast values were extracted from each ROI for each participant using the MarsBaR SPM toolbox (Brett et al., 2002), and contrast values were then averaged across ROIs for each DMN subnetwork and for the whole MD network. Additionally, the same contrasts were examined in a whole brain voxelwise analysis, thresholded at p < 0.05 corrected for multiple comparisons using the false discovery rate (FDR).

## 3 Results

### 3.1 Behavioural performance

Average accuracy was 98.3%, and average RT 0.95s. Independent samples t-tests showed no significant effect of 6-choice domain (6-choice animal or 6-choice word) (accuracy: t(40) = 1.97, p = 0.055, RT: t(40) = 1.07, p = 0.290) so results are collapsed across these groups.

**Fig. 3:**
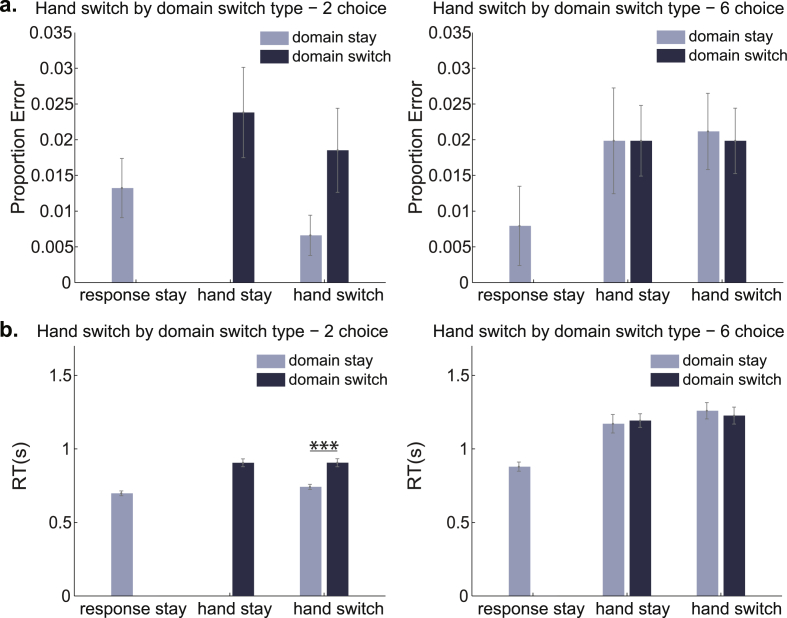
Mean proportion of errors (a) and reaction times (b) for trials of each domain switch type by response switch type, plotted separately for 2-choice and 6-choice conditions. Significant 2-tailed paired t-tests between domain switch types are indicated with *** ​= ​p ​< ​0.01. Error bars show standard error of the mean between participants.

Fig. 3 shows error rate and reaction time for domain-switch type by response-switch type for 2-choice and 6-choice trials separately. Error rate was low across all conditions, while reaction times for 6-choice trials appeared slower than 2-choice trials.

To test for an effect of choice number we performed paired-samples t-tests. To ensure equal numbers of domain switch trials and hand switch trials in 2 and 6 choice measures, we selected the four trial types that were present across both choice levels: domain stay, response stay; domain switch, hand stay; domain stay, hand switch; domain switch, hand switch (Table 1). We first averaged across trials within each of the four trial types before averaging across switch conditions. Paired-sample t-tests showed that responses were significantly faster for 2-choice trials than 6-choice trials (t(41) = 10.05, p < 0.001) but there was no effect of choice number on accuracy (t(41) = 0.09, p = 0.926).

To examine domain switch effects while controlling for response switch and choice number effects, we restricted the analysis to hand switch trials only (see Table 1). A two-way ANOVA with within-subject factors of choice number (2,6) and domain switch type (domain stay, domain switch) was performed for accuracy and reaction time data. For accuracy, there were no significant effects of choice number (F(1,41) = 2.78, p = 0.103), domain switch type (F(1,41) = 1.40, p = 0.243) or an interaction (F(1,41) = 2.46, p = 0.124). For reaction time, there was a significant main effect of choice number (F(1,41) = 72.03, p < 0.001), domain switch type (F(1,41) = 21.79, p < 0.001) and a significant interaction (F(1,41) = 24.36, p < 0.001). Paired-samples t-tests showed responses were significantly faster for domain stay compared to domain switch trials in the 2-choice condition (t(41) = 7.00, p < 0.001) but not the 6-choice condition (t(41) = 1.30, p = 0.201).

In summary, behavioural results show that the manipulation of retrieval difficulty by choice number was effective, and that switches of stimulus domain increased RT in the 2-choice but not the 6-choice conditions.

### 3.2 ROI analysis

**Fig. 4:**
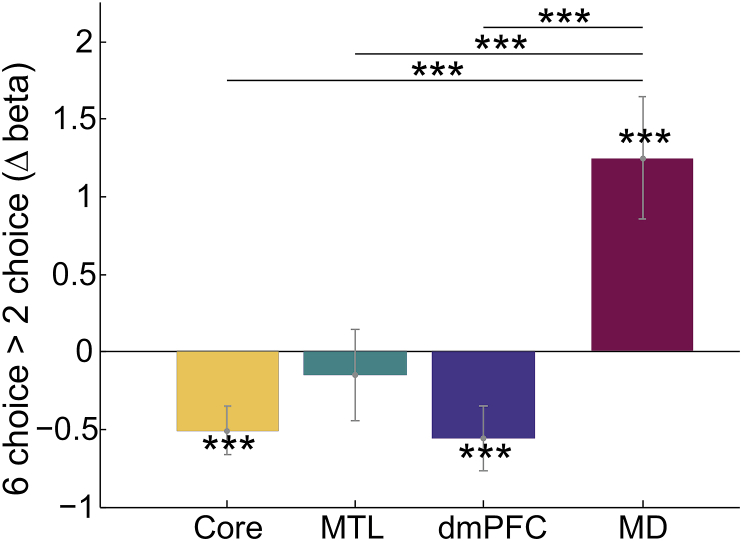
Contrasts of choice number in each DMN subnetwork and the MD network. Significant changes in activity with choice number as well as paired t-tests between subnetworks are indicated with * ​= ​P ​< ​0.05, ** ​= ​p ​< ​0.02 and *** ​= ​p ​< ​0.01. Error bars show standard error of the mean between participants.

To examine the effects of retrieval difficulty, our primary analysis compared brain activity for 2- and 6-choice tasks. For this purpose, we used average beta values over the four trial types that were present across both choice levels: domain stay, response stay; domain switch, hand stay; domain stay, hand switch; domain switch, hand switch (Table 1). Differences in mean beta value for 2- and 6-choice tasks are shown in Fig. 4.

**Supplementary Fig. 1:**
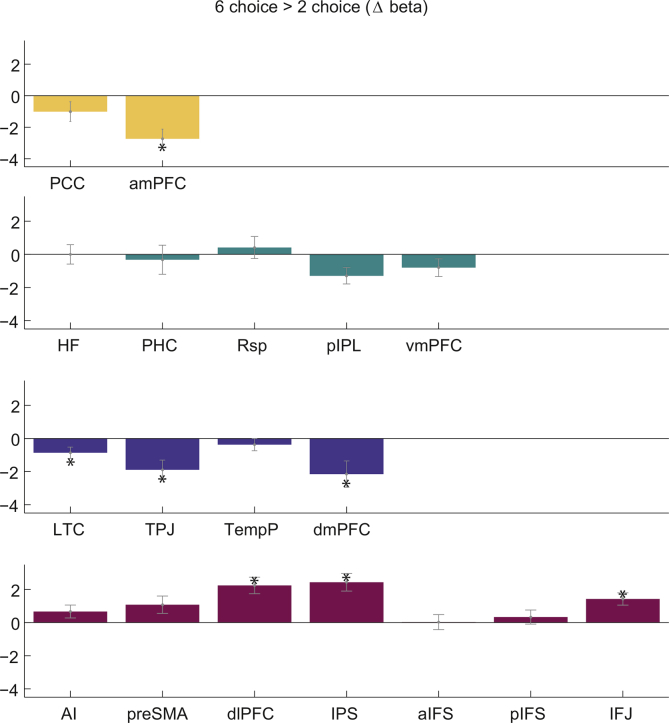
Contrasts of choice number for individual regions of interest within in each DMN subnetwork and the MD network. Significant changes in activity with choice number are indicated with an asterisk (p < 0.05, corrected for the number of regions within each (sub)network by Holm-Bonferroni). Error bars show standard error of the mean between participants. Yellow = core DMN, green = MTL DMN, blue = dmPFC DMN, purple = MD. PCC = posterior cingulate cortex, amPFC = anteromedial prefrontal cortex, HF = hippocampal formation, PHC = parahippocampus, Rsp = retrosplenial cortex, pIPL = posterior inferior parietal lobe, vmPFC = ventromedial prefrontal cortex, LTC = lateral temporal cortex, TPJ = temporo-parietal junction, Temp-Pole = temporal pole, dmPFC = dorsomedial prefrontal cortex, AI = anterior insula, preSMA = pre-supplementary motor area, dlPFC = dorsolateral pre-frontal cortex, IPS = inferior parietal sulcus, aIFS = anterior inferior frontal sulcus, pIFS = posterior inferior frontal sulcus, IFJ = inferior frontal junction.

Results were clear cut. For DMN regions, activation was greater in 2-choice than in 6-choice. This difference was significant in core and dmPFC subnetworks (core: t(41) = 3.16,p < 0.01, dmPFC: t(41) = 2.70,p < 0.01). In the MTL subnetwork, there was no significant effect of choice number (t(41) = 0.78,p = 0.44). These results rule out the hypothesis that DMN activity increases with the number of choice alternatives during rule retrieval. In contrast, MD regions showed significantly greater activity for the more difficult 6-choice compared to 2-choice trials (t(41) = 3.17,p < 0.01). Additional t-tests revealed significant differences in contrast values between MD network regions and all three DMN subnetworks (core: t(41) = 4.54,p < 0.001; MTL: t(41) = 3.43,p < 0.001; dmPFC: t(41) = 5.16,p < 0.001). A finer breakdown by individual regions within each (sub)network showed largely consistent results, though with variable significance across regions (see Supplementary Fig. 1).

**Fig. 5:**
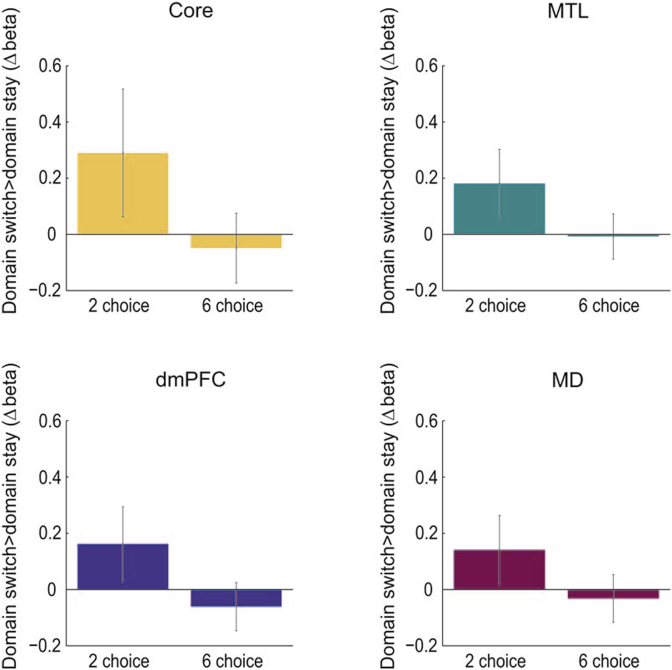
Contrasts of domain switch effects by choice number in each DMN subnet-work and the MD network. No significant (P < 0.05) effects of domain switch type were found. Paired t-tests showed no significant differences between domain switch effects for 2-choice and 6-choice trials. Error bars show standard error of the mean between participants.

**Supplementary Fig. 2:**
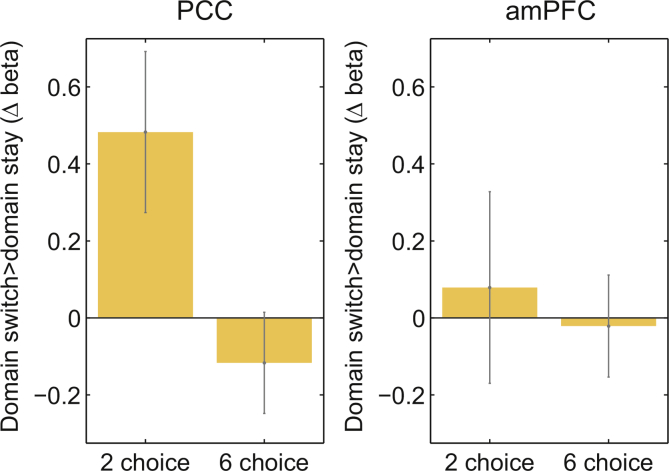
Contrasts of domain switch effects by choice number for individual ROIs within the Core DMN. After correcting for multiple comparisons within subnetworks using the Holm-Bonferroni correction, t-tests showed no significant effects of domain switch type for either 2-choice or 6-choice trials. Paired t-tests also showed no significant differences between domain switch effects for 2-choice and 6-choice trials. Error bars show standard error of the mean between participants. PCC = posterior cingulate cortex, amPFC = anteromedial prefrontal cortex.

**Supplementary Fig. 3:**
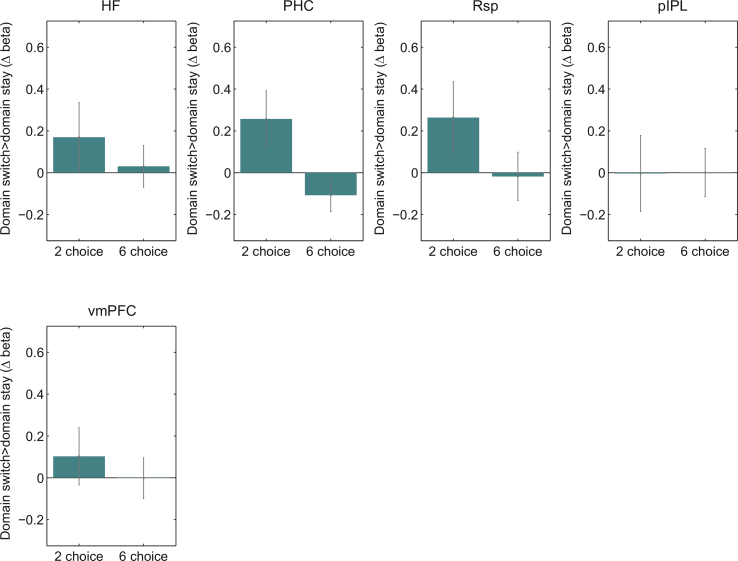
Contrasts of domain switch effects by choice number for individual ROIs within the MTL DMN. After correcting for multiple comparisons within subnetworks using the Holm-Bonferroni correction, t-tests showed no significant effects of domain switch type for either 2-choice or 6-choice trials. Paired t-tests also showed no significant differences between domain switch effects for 2-choice and 6-choice trials. Error bars show standard error of the mean between participants. HF = hippocampal formation, PHC = parahippocampus, Rsp = retrosplenial cortex, pIPL = posterior inferior parietal lobe, vmPFC = ventromedial prefrontal cortex.

**Supplementary Fig. 4:**
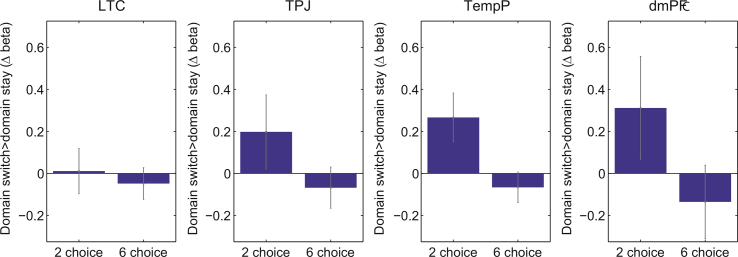
Contrasts of domain switch effects by choice number for individual ROIs within the dmPFC DMN. After correcting for multiple comparisons within subnetworks using the Holm-Bonferroni correction, t-tests showed no significant effects of domain switch type for either 2-choice or 6-choice trials. Paired t-tests also showed no significant differences between domain switch effects for 2-choice and 6-choice trials. Error bars show standard error of the mean between participants. LTC = lateral temporal cortex, TPJ = temporo-parietal junction, Temp-Pole = temporal pole, dmPFC = dorsomedial prefrontal cortex.

**Supplementary Fig. 5:**
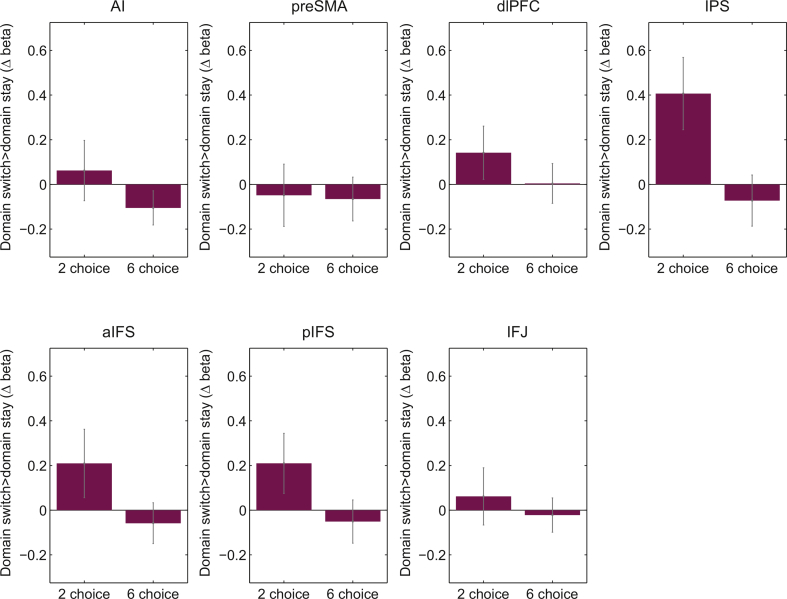
Contrasts of domain switch effects by choice number for individual ROIs within the MD network. After correcting for multiple comparisons within subnetworks using the Holm-Bonferroni correction, t-tests showed no significant effects of domain switch type for either 2-choice or 6-choice trials. Paired t-tests also showed no significant differences between domain switch effects for 2-choice and 6-choice trials. Error bars show standard error of the mean between participants. AI = anterior insula, preSMA = pre-supplementary motor area, dlPFC = dorsolateral pre-frontal cortex, IPS = inferior parietal sulcus, aIFS = anterior inferior frontal sulcus, pIFS = posterior inferior frontal sulcus, IFJ = inferior frontal junction.

We next examined effects of domain switching. For this purpose, we compared mean beta values on domain switch vs domain stay trials, restricting analysis just to hand switch trials (see Table 1). A two-way ANOVA with within-subject factors of choice number (2,6) and domain switch type (domain stay, domain switch) for each (sub)network type was constructed. Reflecting the previous analyses, core, dmPFC and MD (sub)networks showed a significant main effect of choice number (core: F(1,41) = 6.20, p < 0.02; dmPFC: F(1,41) = 8.02, p < 0.01; MD: F(1,41) = 11.55, p < 0.01). However, no subnetworks showed a significant main effect of domain switch type (Core: F(1,41) = 1.13, p = 0.293; MTL: F(1,41) = 1.82, p = 0.185; dmPFC: F(1,41) = 0.571, p = 0.454; MD: F(1,41) = 0.610, p = 0.439) or a significant interaction (Core: F(1,41) = 1.37, p = 0.248; MTL: F(1,41) = 1.39, p = 0.251; dmPFC: F(1,41) = 1.56, p = 0.219; MD: F(1,41) = 1.18, p = 0.284) (see Fig. 5). Separate t-tests also showed no significant effect of switching for either 2- or 6-choice tasks. When the analysis was repeated collapsing across all DMN regions rather than separating by subnetwork, again there was a significant main effect of choice number (F(1,41) = 5.45, p < 0.05), but no main effect of domain switch type (F(1,41) = 1.16, p = 0.289) nor interaction (F(1,41) = 2.19, p = 0.146). Supplementary analyses for individual regions also did not show significant domain switch effects or interaction effects (see Supplementary Figs. 2–5).

### 3.3 Whole brain analysis

**Fig. 6:**
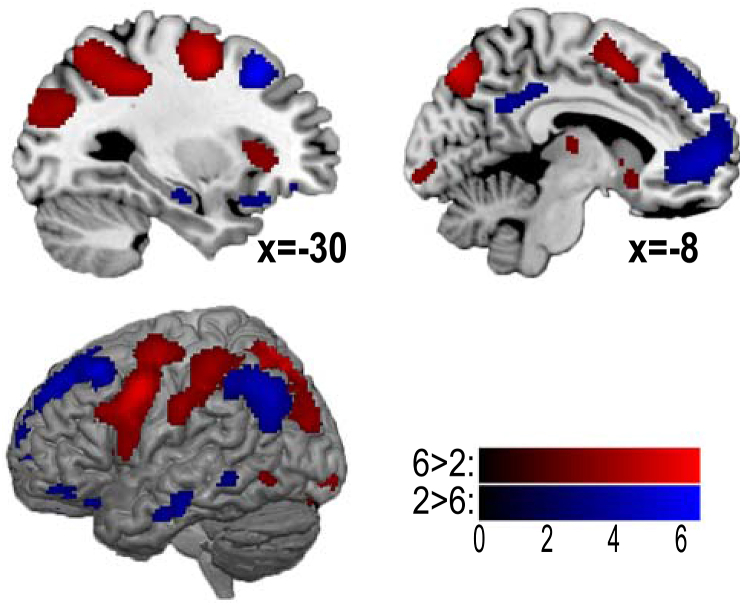
Whole brain contrast values for choice number presented with an FDR corrected threshold of p < 0.05, plotted in MNI space. The red colour scale represents 6 choice>2 choice. The blue colour scale represents 2 choice> 6 choice. Brain render shows search depth of 12 voxels. Medial slices show x coordinate values.

Fig. 6 shows the results of the whole brain analysis for the contrasts of 6-choice>2-choice and 2-choice>6-choice, thresholded at p < 0.05, corrected for false discovery rate. In line with the ROI analysis, several MD network regions were found to be more active for 6-choice compared to 2-choice trials, including the anterior insula, inferior prefrontal and dorsal prefrontal cortex, intraparietal sulcus and pre-supplementary motor area. Stronger activity in the 6-choice task was also seen in a number of other regions, including precuneus, sensorimotor cortex, occipitoparietal cortex and regions in the basal ganglia. In contrast, greater activity in 2-choice than 6-choice was prominent across much of the DMN, including dorsomedial prefrontal, anteromedial prefrontal, ventromedial prefrontal and lateral temporal cortex, posterior cingulate, hippocampus, and temporo-parietal junction.

At the whole brain level, we found no significant effects of domain switch vs domain stay for either 2 choice or 6 choice tasks, or when combined across choice number.

## 4 Discussion

The primary aim of this study was to test whether increased difficulty of rule retrieval is associated with increased DMN activity. To achieve this, we manipulated the number of alternative rules in a task set. This manipulation had a large effect on behaviour with participants performing significantly slower for 6-choice trials compared to 2-choice trials. However, DMN activity, at least in core and dmPFC regions, showed deactivation with increased retrieval difficulty. This rules out the retrieval difficulty hypothesis and instead matches many cases of deactivation with increasing task difficulty (McKiernan et al., 2003; Fransson, 2006; Leech et al., 2011). Other studies have also looked at manipulating the number of alternatives or response mapping retrieval demands (Badre and D’Esposito, 2007; Crittenden and Duncan, 2012). These studies found increased activity in dorsal premotor cortex, intraparietal sulcus and pre-supplementary motor area for increased number of response buttons and do not report any DMN-related activity. Replicating these findings, we found that activity in MD regions increased with retrieval difficulty. This is consistent with many previous results showing that MD activity increases with task difficulty (Duncan, 2013; Fedorenko et al., 2013) and working memory demands (Cohen et al., 1997; Owen et al., 2005).

As stated in the introduction, DMN activity has been linked to different aspects of retrieval (e.g.Smallwood et al., 2013; Konishi et al., 2015; Murphy et al., 2018), but especially to episodic recollection. Tulving and Murray (1985) outlined three key properties of episodic memory: a subjective sense of time (a feeling of mental time travel), connection to the self (self-relevance), and autonoetic consciousness (the cognitive ability to mentally project oneself to an imagined time and place). Particularly supporting these ideas, DMN regions have been found to show increased activity with increased self-relevance, vividness of the remembered episode, and self-projection (Andrews-Hanna et al., 2010; Richter et al., 2016). Rule retrieval is not deeply personal, nor does it require mental projection back to a time and place. Perhaps for these reasons, it seems that the role of the DMN in memory retrieval does not extend to retrieval of task rules.

Given the negative relationship between DMN activity and retrieval demand, in isolation the current results could be interpreted in line with the common view of the DMN as task-negative, showing deactivation with increasing demand of an externally-focused task. However, a large body of work suggests that the relationship between the DMN and task related processes is more complex. For example, during a sustained attention task, DMN activity was positively related to a state of reaction time stability in which participants made fewer errors, however, within this state, further increases in DMN activation were predictive of errors (Esterman et al., 2012). Similarly, Sormaz et al. (2018) found that DMN activity reflected the level of detail during working memory maintenance in a 1-back task despite reduced univariate activity compared to the 0-back version (see also Murphy et al., 2019b). Further studies have also found that DMN regions represent task information. For example, Smith et al. (2018) found that patterns of activity in DMN regions could distinguish between upcoming task domains (see also Crittenden et al., 2015). Similarly, Schuck et al., 2016 found that DMN activity patterns could be used to decode between different task contexts (house or face judgements). Thus it seems that many aspects of the DMN response are associated with active task processing, even in the absence of overall increases in univariate activity.

A secondary goal of this research was to see whether domain switches cause increased DMN activation in a much simpler task setting than used previously (Crittenden et al., 2015; Smith et al., 2018). In this task the results do not show domain switch effects in DMN regions. Of course, this lack of DMN switch effect is not unusual: DMN domain switch effects are not typically reported in experiments when one is switching between just two tasks (Kimberg et al., 2000; Yeung et al., 2006). The lack of switch effects in the current study may help understand the switch-related DMN activity found in previous tasks (Crittenden et al., 2015; Smith et al., 2018). Perhaps this activity is stronger in a more elaborate cognitive setting, in which parts of the task are hierarchically chunked and there is switching from one large chunk to another.

Unlike core and MTL DMN subnetworks, the dmPFC subnetwork more consistently shows activity decreases with difficulty, even during large domain switches (Crittenden et al., 2015; Smith et al., 2018; McKiernan et al., 2003; Fransson, 2006; Leech et al., 2011). In the present study, the effect of rule retrieval was observed in core and dmPFC subnetworks, but not the MTL subnetwork. It remains to be understood what specific task demands cause these dissociations between subnetwork regions as opposed to their usual functional correlation, although some principles have been proposed (e.g. Andrews-Hanna et al., 2010; 2012). Evidence from Poerio et al. (2017) also shows functional dissociations between DMN subnetworks. These researchers found that connectivity between superior frontal gyrus (including the dmPFC) and MTL regions was negatively related to task performance requiring external engagement, including the Tower of London task and encoding facts in stories. These results suggest that good performance in tasks of this sort may require a trade-off between different DMN subnetwork regions.

In summary, we find that the difficulty of rule retrieval cannot satisfactorily explain the previous findings of Crittenden et al. (2015) and Smith et al. (2018). Instead, we conclude that these DMN activations are a genuine effect of large switches, at least in complex, hierarchical settings. If the DMN represents context, as much evidence suggests (Zacks et al., 2007; Ranganath and Ritchey, 2012; Baldassano et al., 2016; Milivojevic et al., 2016; Chen et al., 2017), these findings (Crittenden et al., 2015; Smith et al., 2018) indicate that this context may be reawakened with a large cognitive switch. Further work should address the detail of DMN context representations, and their fluctuations during cognitive maintenance and transition.

## Data and code availability statement

Data and code is available upon direct request to the corresponding author.
